# Microorganisms associated with access-associated bacteremia in hemodialysis outpatients in Saudi Arabia

**DOI:** 10.1186/1753-6561-5-S6-P209

**Published:** 2011-06-29

**Authors:** HH Balkhy, A El-Saed, F Al Hejaili, M Sallah, B Abu Khzam, A Sayyari

**Affiliations:** 1 KAMC, Riyadh, Saudi Arabia

## Introduction / objectives

The number of patients on hemodialysis in Saudi Arabia doubled over the last decade. Monitoring organisms associated with access-associated bacteremia is necessary for empirical case-management.

## Methods

A prospective surveillance study for all end-stage kidney disease patients at KAMC in Riyadh, SA. The same methodology and definitions applied by US National Healthcare Safety Network (NHSN) centers was used to allow benchmarking. Organism rates at KAMC were compared to those reported by NHSN.

## Results

Out of 198 positive blood cultures recorded during the study, 174 (88%) were access-associated bacteremia. The majority (89%) of these bacteremias were cause by a single organism. Out of the 190 organism identified 51.6% gram-negatives, 39.5% gram-positives, 8.5% skin contaminants, and 0.5% fungi. There was no significant difference of the organism distribution between those with catheter and those with AV fistula or graft (p=0.973). Staphylococcus aureus (17.9%) and Enterococcus sp (16.8%) were the most common gram-positives. Klebsiella sp (13.2%), Enterobacter sp (11.6%), and Pseudomonas aeruginosa (6.8%) were the most common gram-negatives. Compared to NHSN centers, KAMC had significantly higher gram negative (47.9% vs 21.3%, p<0.001), lower skin contaminants (13.0% vs 43.1%, p<0.001), but similar gram-positives (39.7% vs 34.2%, p=0.171).

## Conclusion

In KAMC hemodialysis patients known of their high rates of permanent catheter and access-associated bacteremia, gram negative rods were the most common organisms identified. The causes of such finding whether colonization pattern, catheter care, empirical antimicrobials, or patient cormorbidities need to be delineated in future studies.

## Disclosure of interest

None declared.

